# Relationship between vitamin D and chronic spontaneous urticaria: a systematic review

**DOI:** 10.1186/s13601-018-0234-7

**Published:** 2018-12-04

**Authors:** Papapit Tuchinda, Kanokvalai Kulthanan, Leena Chularojanamontri, Sittiroj Arunkajohnsak, Sutin Sriussadaporn

**Affiliations:** 1 Department of Dermatology, Faculty of Medicine Siriraj Hospital, Mahidol University, 2 Wanglang Road, Bangkoknoi, Bangkok, 10700 Thailand; 2 Division of Endocrinology and Metabolism, Department of Medicine, Faculty of Medicine Siriraj Hospital, Mahidol University, Bangkok, Thailand

## Abstract

**Background:**

Vitamin D has been reported to be associated with many allergic diseases. There are a limited number of the studies of vitamin D supplementation in patients with chronic spontaneous urticaria (CSU). This study aims to study the relationship between vitamin D and CSU in terms of serum vitamin D levels, and the outcomes of vitamin D supplementation.

**Methods:**

A literature search of electronic databases for all relevant articles published between 1966 and 2018 was performed. The systematic literature review was done following Preferred Reporting Items for Systematic Reviews and Meta-analysis recommendations.

**Results:**

Seventeen eligible studies were included. Fourteen (1321 CSU cases and 6100 controls) were concerned with serum vitamin D levels in CSU patients. Twelve studies showed statistically significant lower serum vitamin D levels in CSU patients than the controls. Vitamin D deficiency was reported more commonly for CSU patients (34.3–89.7%) than controls (0.0–68.9%) in 6 studies. Seven studies concerned with vitamin D supplementation in CSU patients showed disease improvement after high-dosages of vitamin D supplementation.

**Conclusion:**

CSU patients had significantly lower serum vitamin D levels than the controls in most studies. However, the results did not prove causation, and the mechanisms were not clearly explained. Despite the scarcity of available studies, this systematic review showed that a high dosage of vitamin D supplementation for 4–12 weeks might help to decrease the disease activity in some CSU patients. Well-designed randomized placebo-controlled studies are needed to determine the cut-off levels of vitamin D for supplementation and treatment outcomes.

## Background

Chronic spontaneous urticaria (CSU) is defined as the occurrence of spontaneous wheals, angioedema, or both for more than 6 weeks [1]. Recommended first-line treatment is modern, second-generation H_1_-antihistamines. For refractory patients, a short course of systemic corticosteroids, omalizumab or ciclosporin is recommended [1].

Vitamin D, a fat-soluble vitamin, exists in two forms: D_2_ (ergocalciferol) and D_3_ (cholecalciferol) [2]. The human body gains it from the diet and sunlight. Vitamin D_2_ has been found in some mushrooms, e.g., shiitake mushrooms and button mushrooms. Vitamin D_3_ is commonly found in halibut, mackerel, eel, salmon, beef liver, and egg yolks [3]. Within the human body, only the skin can produce vitamin D_3_. Ultraviolet B radiation (wavelength, 290–315 nm) converts 7-dehydrocholesterol in the skin to previtamin D_3_, which is rapidly converted to vitamin D_3_. Vitamins D_2_ and D_3_ from diets and vitamin D_3_ from skin photobiosynthesis are initially metabolized by the liver enzyme 25-hydroxylase (CYP2R1) to 25-hydroxyvitamin D (25(OH)D), the major circulating metabolite which is commonly used for evaluation of vitamin D status. The 25(OH)D is metabolized in the kidneys by the enzyme 25-hydroxyvitamin D-1α-hydroxylase (CYP27B1) to 1,25-dihydroxyvitamin D (1,25(OH)_2_D), the most biologically active form of vitamin D [2].

Vitamin D plays a major role in mineral homeostasis [2]. Besides its role in bone physiology, it also has a role on cutaneous immunity by binding to its nuclear receptors and plasma membrane receptors of epithelial cells, and to various cells such as mast cells, monocytes, macrophages, T-cells, B-cells, and dendritic cells [4, 5]. In the innate immune system, vitamin D contributes to improving antimicrobial defenses by stimulating the expression of antimicrobial peptides such as cathelicidin and human β-defensin [6]. In the adaptive immune system, in vitro study showed that physiologic (in vivo) concentration of 25(OH)D_3_ in serum-free medium can activate T cells to express CYP27B1 and then convert 25(OH)D_3_ to 1,25(OH)_2_D_3_. (active form of vitamin D) [7]. Vitamin D can suppress dendritic cell maturation and inhibits Th1 cell proliferation by decreasing Th1 cytokine secretion. It also induces hyporesponsiveness by blocking proinflammatory Th17 cytokine secretion and decreasing interleukin (IL)-2 production from regulatory T (Treg) cells. It inhibits B-lymphocyte function resulting in the reduction of immunoglobulin E production [8, 9]. Moreover, vitamin D has influences on the proliferation, survival, differentiation, and function of mast cells [5, 10].

The vitamin D binding protein (VDBP) and vitamin D receptor (VDR) are two proteins that influence the biological actions. VDBP is the main carrier protein in the circulation. Group-specific component (GC) is the gene that encodes VDBP [11]. Genetic polymorphism in the GC gene influences the concentration of VDBP and its affinity for vitamin D. Regarding VDR, the binding of VDR to vitamin D results in epigenetic modification and transcription of various specific genes [12]. The human VDR gene is located in chromosome 12. Polymorphism in the VDR gene has been shown to alter VDR functions that affect vitamin D activities [13]. Among the VDR polymorphisms, the SNPs rs1544410 and rs2228570 are frequently studied in association with allergic diseases. However, Nasiri–Kalmarzi et al. reported no significant correlation between the VDR rs2228570 and VDBP rs7041 SNPs and the development of chronic urticaria (CU), although they found a positive correlation between serum VDBP and the progression of CU. They concluded that alteration of the vitamin D pathway at the gene and protein levels may be a risk factor for the progression of CU [14].

There have been reports of an association between vitamin D and allergic diseases, such as food allergies, rhinosinusitis, recurrent wheeze, asthma, atopic dermatitis, and CSU [15–17]. Some studies have shown that vitamin D is involved in the etiopathogenesis of CSU, while other studies have demonstrated clinical improvement in CSU with vitamin D supplements. However, there are a limited number of studies on this issue, and their results are inconsistent. [14, 18–33].

We performed a systematic review to examine the serum vitamin D levels in patients with CSU. Data concerning vitamin D supplementation in the CSU patients were also studied to determine whether supplementation impacts treatment outcomes.

## Methods

### Search strategy and selection criteria

This systematic review adhered to Preferred Reporting Items for Systematic Reviews and Meta-analysis recommendations (PRISMA).

**Fig. 1:**
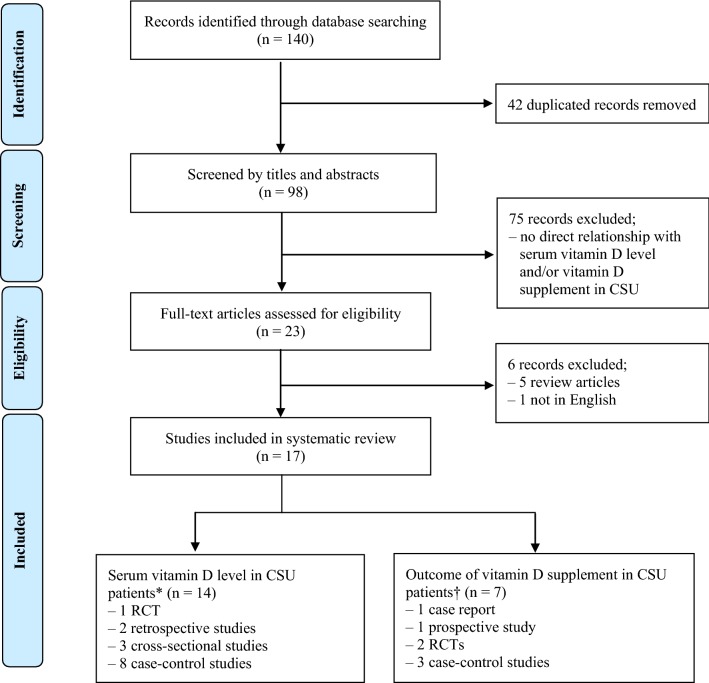
Flow diagram of literature review in this study. Seventeen studies met the inclusion criteria and were included in our systematic review. *Of the 14 studies, the relation between serum vitamin D level and CSU were assessed [14, 18, 20–25, 28–33]. ^†^In 7 studies, various severity assessment were used to evaluate the effect of vitamin D supplementation in CSU patients [19, 24–27, 31, 32]. *CSU* chronic spontaneous urticaria

A literature search of electronic databases (PubMed, Scopus, Web of Science, MEDLINE, The Cochrane Library, and CINAHL) for all relevant articles published between Jan 1, 1966, and September 30, 2018 was conducted using the search term “chronic urticaria and vitamin D or 25(OH)D insufficiency or deficiency or 1,25 (OH)_2_ vitamin D insufficiency or deficiency” The titles and abstracts of the articles identified in the search were screened by two independent reviewers (KK and SA) for eligibility based on the inclusion criterion. Full texts were then obtained and assessed for eligibility by those two reviewers (KK and SA). A further manual search of the references cited in the selected articles was subsequently performed to identify any relevant studies that might have been missed in the initial search. Finally, all yielded relevant reports were systematically reviewed (Fig. 1).

Any types of publication involving vitamin D in CSU patients were included in our systemic review. The exclusion criteria were: (1) articles that were not published in English; (2) duplicated publications; (3) studies published only in abstract form; and (4) continuous medical education (CME) and review articles.

### Assessment of risk of bias in included studies

Two investigators (KK and SA) assessed the risk of bias of the eligible studies included in this systematic review. We used Cochrane Collaboration’s tool to assess the risk of bias in randomized controlled trials (RCTs). The Risk Of Bias In Non-randomized Studies-of Interventions (ROBINS-I) tool was used to assess the risk of bias in non-RCT studies.

### Data extraction for serum vitamin D levels and CSU

The search strategies were mainly used to identify vitamin D levels, and to compare the levels found in CSU patients and controls. Serum vitamin D levels are mostly reported in the form 25(OH)D. After the eligible full-text articles were reviewed and the relevant data reported in those articles were further searched, the following information was extracted from each: the first author, year of publication, type of study, number and characteristics of the population, number of cases and controls, method of vitamin D measurement, type (form) and unit of the measured serum vitamin D, vitamin D levels in case and control groups, and study outcomes. Information was completely and carefully extracted from the eligible articles.

### Data extraction for treatment or supplementation of vitamin D

We also examined whether vitamin D supplementation has an impact on the outcomes of urticaria treatment. All relevant data were extracted, namely, the first author, year of publication, type of study, number and characteristics of cases and/or controls, form, dosage and duration of vitamin D treatment, assessment duration, methods and parameters for outcome measurement, vitamin D status at baseline and after vitamin D treatment, and treatment outcomes.

## Results

### Literature search

The detailed steps of the literature search are illustrated in the flow chart at Fig. 1. A total of 140 potentially relevant studies were found. The titles and abstracts of these articles were reviewed. Of the 117 excluded studies, 42 were removed due to duplication and 75 were irrelevant; the remainder (23 studies) were screened for full text review. According to the inclusion and exclusion criteria, 5 review articles were excluded, and 1 study was excluded because it had not been published in English. The full texts of the remaining 17 studies were extensively reviewed, and all were finally included [14, 18–33].

### Characteristics of included studies

The 17 studies were published during the period 2010–2018 [14, 18–33]. The main characteristics of the studies were summarized into two issues: serum vitamin D levels in CSU patients, and outcomes of vitamin D supplementation in CSU patients.

### Risk of bias

Three RCTs in our systematic review were estimated mainly at low risk. The majority of the non-RCT studies had a low risk of bias according to ROBIN-I assessment.

### Serum vitamin D levels in CSU patients

Fourteen studies were concerned with serum vitamin D levels in CSU patients. There were 1 RCT [32], 3 cross-sectional studies [20, 22, 33], 8 case–control studies [14, 18, 21, 23–25, 28, 31], and 2 retrospective reviews [29, 30] (Table 1). All studies drew upon data from a total of 7421 participants, with 1321 patients with CSU and 6100 controls, including 5456 healthy controls, and 25 cases of allergic rhinitis controls. The remaining 619 participants were 593 acute urticaria patients and 26 atopic dermatitis patients. Statistical analyses for meta-analysis were not performed due to the substantial heterogeneity of the reported data.

**Table 1:** Serum vitamin D levels in CSU patients

Study, year	Study size/population	Vitamin D data						Outcome
		Methods	Units	Serum 25(OH)D levels				
				CSU		Controls		
*Cross-sectional study*								
Chandrashekar et al. [20]	45 CSU 45 age-, sex-matched healthy controls	ELISA kit (Euroimmun AG, Lubeck, Germany)	ng/mL	12.7 ± 2.7 (mean ± SD)		24.3 ± 13.5 (mean ± SD) (p < 0.0001)		Significant lower vitamin D levels among chronic urticaria patients and controls Significant lower vitamin D levels in APST positive group (11.1 ± 2.1 ng/mL) compared with APST negative group (15.1 ± 1.3 ng/mL) (p < 0.0001) Significant negative correlation between vitamin D levels and USS, IL-17, TGF-β1 and ESR (p < 0.0001)
Lee et al. [22]	57 CSU 567 acute urticaria 3159 controls	ND	ng/mL	22.9 ± 4.9 (mean ± SD)		Acute urticaria; 20.5 ± 5.1 (mean ± SD) (p = 0.069) Controls; 20.0 ± 5.1 (mean ± SD) (p = 0.124)		The study was conducted in children No significant difference in the 25(OH)D levels between CSU patients and acute urticaria patients and controls (p = 0.183)
Rather et al. [33]	110 CSU 110 age-, sex-matched healthy controls	Chemiluminescence method/kit method (Siemens, USA)	ng/mL	19.6 ± 6.9 (mean ± SD)		38.5 ± 6.7 (mean ± SD) (p < 0.001)		Significant lower vitamin D levels in CSU patients compared with controls Significant negative correlation between serum vitamin D level and UAS (p < 0.001) Significant lower vitamin D levels in CSU patients with the ASST positive subjects than in the ASST negative subjects (p < 0.001) No significant correlation between vitamin D level and duration of the disease
*Case–control study*								
Thorp et al. [28]	25 CSU 25 allergic rhinitis controls	ND	ng/mL	29.4 ± 13.4 (mean ± SD)		39.6 ± 14.7 (mean ± SD) (p = 0.016)		Significantly reduced vitamin D levels in CSU patients compared with controls No correlation of vitamin D levels and duration, severity of disease, ASST or thyroid autoantibody testing No significant difference in the proportion of vitamin D deficiency between CSU groups and controls
				*Vitamin D status*				
				Vitamin D deficiency (< 30 ng/mL)				
				48% (12/25)		28% (7/25) (p = 0.24)		
Abdel-Rehim et al. [18]	22 CSU 20 age- and sex-matched controls *Disease severity* 8 (36.4%): moderate urticaria (UAS7 = 16–27) 14 (63.6%): severe urticaria (UAS7 = 28–42)	ELISA kit (Immundiagnostik AG, Bensheim, Germany)	nmol/L	28.4 ± 9.09 (mean ± SD)		104.5 ± 76.8 (mean ± SD) (p < 0.01)		Significantly lower vitamin D levels among patients in comparison to controls Negative correlation between vitamin D levels and IgE levels (r = 0.45, p < 0.05) No association between vitamin D levels and duration and the severity of the disease
Grzanka et al. [21]	35 CSU 33 age-, sex- and BMI (< 30) matched healthy controls	An automated direct electrochemiluminescence immunoassay (Elecsys, Roche Diagnostic, Mannheim Germany)	ng/mL	26.0 (median)		31.1 (median) (p = 0.017)		Significantly lower serum 25(OH)D concentration in CSU group compared with the control subjects No significant differences in serum 25(OH)D concentration between the mild and moderate-severe symptoms patients Slightly significantly lower 25(OH)D concentrations in moderate-severe CSU than those of the controls (22.6 vs 31.1 ng/mL, p = 0.048) No significant difference in vitamin D levels between mild CSU and healthy control subjects Significantly higher proportion of vitamin D deficiency (< 20 ng/mL) in patients with CSU than in the normal population No significant difference in the prevalence of vitamin D insufficiency (20–29 ng/mL) between CSU patients and the normal subjects No significant correlations between serum concentration of CRP and 25(OH)D levels No significant difference in serum 25(OH) concentrations and ASST testing
				*Vitamin D status*				
				Vitamin D insufficiency (20–< 30 ng/mL)				
				31.4% (11/35)		39.4% (13/33) (p = 0.41)		
				Vitamin D deficiency (< 20 ng/mL)				
				31.4% (11/35)		6% (2/33) (p = 0.025)		
				Severe vitamin D deficiency (< 10 ng/mL)				
				2.9% (1/35)		0% (0/33) (p = 0.52)		
Movahedi et al. [23]	114 CSU 187 sex- and age-matched healthy controls	Enzyme immunoassay method (EIA) (Immunodiagnostic system; IDS (LTD), UK)	ng/mL	15.8 ± 1.5		22.6 ± 1.6 (p = 0.005)		Significantly lower serum 25(OH)D concentration in CSU group compared to healthy subjects No significant differences in vitamin D levels between autoimmune chronic urticaria patients and the control group (p = 0.11) Significant association between vitamin D deficiency and increased susceptibility to CSU (p = 0.001) A 2.4-fold (95% CI 1.4–4) risk of having CSU in individuals with vitamin D deficiency (< 20 ng/ml) Significantly lower levels of vitamin D in patients with longer duration of urticaria symptoms (> 24 h) (p = 0.046) A significant positive correlation between vitamin D levels and UAS (r = 0.2, p = 0.042) No significant relationship between IgE levels and vitamin D levels
				*Vitamin D status*				
				Vitamin D sufficiency				
				8.8% (10/114)		26.2% (49/187)		
				Vitamin D insufficiency (20–30 ng/mL)				
				15.8% (18/114)		16.6% (31/187)		
				Vitamin D deficiency (< 20 ng/mL)				
				75.4% (86/114)		57.2% (107/187)		
Rasool et al. [25] (Randomized case–control)	147 moderate-severe CSU 130 healthy controls	Enzyme immunoassay	ng/mL	17.87 ± 1.22 (mean ± SEM)		27.65 ± 1.65 (mean ± SEM) (p < 0.0001)		Low serum 25(OH)D levels in 91% of CSU patients and 64% of the healthy subjects Significantly lower vitamin D levels in CSU patients compared with controls
				*Vitamin D status*				
				Vitamin D insufficiency (20–30 ng/mL) or Vitamin D deficiency (10–20 ng/mL)				
				91.3%		63.84% (p < 0.0001)		
Boonpiyathad et al. [31] (Prospective case–control)	60 CSU 40 healthy controls	ND	ng/mL	15.0 (7–52) median (min–max)		30.0 (25–46) median (min–max) (p < 0.001)		Significantly lower the median 25(OH)D concentration in the CSU group than the control group Significantly higher patients with vitamin D deficiency (< 20 ng/mL) in the CSU group than the control group (p < 0.001) No association between UAS7 and DLQI scores with 25(OH)D levels Significant correlation between ESR and vitamin D levels (p = 0.001)
				*Vitamin D status*				
				Vitamin D insufficiency (> 20–< 30 ng/mL)				
				28%		45% (p = 0.38)		
				Vitamin D deficiency (< 20 ng/mL)				
				55%		0% (p < 0.001)		
Oguz Topal et al. [24] (Prospective case–control)	58 CSU 45 healthy age-matched controls *Disease severity* 3 (5.2%): mild urticaria (UAS4^a^: 0–8) 15 (25.8%): moderate urticaria (UAS4: 9–16) 40 (68.9%): severe urticaria (UAS4: 17–24)	An automated direct electrochemiluminescence immunoassay (Elecsys, Roche Diagnostic, Mannheim, Germany)	ug/L	All CSU 8.45 (1.1–52.5) median (min–max) (p < 0.001) Mild-moderate CSU 8.95 (3.9–23.0) median (min–max) (p = 0.011) Severe CSU 7.1 (1.1–52.5) median (min–max) (p < 0.001)		15.3 (3.1–61.0) median (min–max)		Significantly lower serum 25(OH)D concentration in total CSU group, mild-moderate CSU group and severe CSU group compared to healthy subjects Significantly higher prevalence of vitamin D deficiency and insufficiency in CSU patients No significant differences in 25(OH)D levels between CSU patients with mild-moderate symptoms and severe symptoms No significant differences between vitamin D-deficient or insufficient group regarding CU-Q2oL and UAS4 scores (p > 0.001) No association between the anti-TG and the anti-TPO autoantibodies and the levels of vitamin D in CSU patients, (p = 0.641 and p = 0.373, respectively) No association between the prevalence of high levels of total IgE and the levels of vitamin D in CSU patients (p = 0.5)
				*Vitamin D status*				
				Vitamin D insufficiency (< 30 μg/L)				
				98.3% (57/58)		86.7% (39/45) (p = 0.041)		
				Vitamin D deficiency (< 20 μg/L)				
				89.7% (52/58)		68.9% (31/45) (p = 0.017)		
Nasiri-Kalmarzi et al. [14]	110 CSU 110 healthy controls	Specific E LISA (Monobind Inc., Lake Forest, CA, USA)	ng/mL	19.26 ± 1.26 (mean ± SEM)		31.72 ± 7.14 (mean ± SEM) (p = 0.006)		Significantly lower serum vitamin D levels in chronic urticaria patients compared to controls Significantly association between decreased levels of serum vitamin and increased susceptibility to chronic urticaria (p = 0.027) Significant negative correlation between vitamin D levels with ASST and UAS (p < 0.001 and p = 0.001, respectively) No significant correlation between vitamin D levels and serum total IgE (p = 0.083) Higher prevalence of vitamin D deficiency or insufficiency in chronic urticaria patients No significant correlation between vitamin D levels and total IgE levels
				*Vitamin D status*				
				Vitamin D deficiency or insufficiency				
				58.02%		48.89%		
*Randomized controlled trial*								
Dabas et al. [32]	241CSU 184 healthy controls	ND	nmol/L	17.47 ± 13.36 (mean ± SD)		22.09 ± 14.06 (mean ± SD) (p = 0.002)		Significantly lower vitamin D level were in CSU patients than in healthy controls No correlation between vitamin D deficiency and sex, ASST, APST, serum IgE, angioedema or disease duration
				*Vitamin D status*				
				Vitamin D sufficiency (> 30 ng/mL)				
				20.91% (23/110)		64.54% (71/110)		
				Vitamin D insufficiency (20–30 ng/mL)				
				15.45% (17/110)		21.82% (24/110)		
				Vitamin D deficiency (< 20 ng/mL)				
				63.64% (70/110)		13.64% (15/110)		
*Retrospective study*								
Woo et al. [29]	72 CSU 26 acute urticaria 26 atopic dermatitis 72 healthy controls	ND	ng/mL^b^	*CSU*	*Acute urticaria*	*Atopic dermatitis*	*Healthy controls*	Both children and adults were enrolled Significantly lower serum 25(OH)D_3_ levels in CSU group compared to those in the other groups Significantly higher proportion of patients with critically low vitamin D levels (< 10 ng/mL) in the CSU group than in acute urticaria, atopic dermatitis, and healthy controls Significant negative associations between the vitamin D levels and urticaria activity score and disease duration (p < 0.001, p = 0.008, respectively) Significantly more critically low vitamin D status in the moderate/severe UAS group than in the mild UAS group (p = 0.03) Significantly lower serum vitamin D levels in subjects with a positive ASST than in subjects with a negative result Significantly higher number of patients with critically low vitamin D in the moderate/severe UAS group than in the mild UAS group (p = 0.03) Significantly lower vitamin D levels in the ASST positive subjects (9.12 ± 4.25 ng/mL) than in the ASST negative subjects (13.33 ± 7.09 ng/mL) (p = 0.034) Significantly higher proportion of those with critically low vitamin D status in the ASST positive group (60%) than in the ASST negative group (32%) (p = 0.021)
				11.86 ± 7.16 (mean ± SD)	14.12 ± 5.56 (mean ± SD) (p = 0.024)	16.12 ± 8.09 (mean ± SD) (p = 0.008)	20.77 ± 9.74 (mean ± SD) (p < 0.001)	
				*Vitamin D status*				
				Sufficiency (≥ 30 ng/mL)				
				2% (2/72)	0%	2%	20% (15/72)	
				Insufficiency (between 20 and 29 ng/mL)				
				10% (7/72)	11%	24%	27% (20/72)	
				Deficiency (< 20 ng/mL)				
				39% (28/72)	63%	46%	45% (32/72)	
				Critically low (< 10 ng/mL)				
				49% (35/72)	26% (6/26) (p < 0.002)	28% (7/26) (p < 0.004)	8% (5/72) (p < 0.001)	
Wu et al. [30]	225 CSU 1321 healthy controls	ND	nmol/L	*CSU*		*Controls*		Significantly higher vitamin D levels in CSU patients than the general population
				51.4 ± 27.03 (mean ± SD)		45.4 ± 24.84 (mean ± SD) (p = 0.001)		

25(OH)D, 25-hydoxyvitamin D; anti-TG, anti-thyroglobulin; anti-TPO, anti-thyroidperoxidase; APST, autologous plasma skin test; ASST, autologous serum skin test; BMI, body mass index; CSU, chronic spontaneous urticaria; CU-Q2oL, chronic urticaria quality of life questionnaire; DLQI, Dermatology Life Quality Index; ELISA, enzyme linked immunesorbent assay; ESR, erythrocyte sedimentation rate; Ig, immunoglobulin; IL, interleukin; ND, not defined; TGF-β1, transforming growth factor β1; UAS, urticaria activity score; USS, urticaria symptom severity

^a^UAS4 (the Urticaria Activity Score over 4 days; (scale 0–6) calculated as the sum of daily average morning and evening scores for itch severity (0, none; 1, mild; 2, moderate; 3, severe) and number of hives (0, none; 1, < 20 hives; 2, 20–50 hives; and 3, > 50 hives)

^b^Serum vitamin D was evaluated as 25(OH)D_3_

The methods used for the measurement of vitamin D varied among the studies (Table 1). All of the studies reported the serum vitamin D level as 25(OH)D except two: one study by Woo et al. [29], which measured 25(OH)D_3_, and Nasiri–Kalmarzi’s study, which did not report the type of vitamin D measured [14]. The units of serum 25(OH)D were reported mainly in ng/mL [14, 20–23, 25, 28, 29, 31, 33], but some studies reported them in µg/L [24] and nmol/L [18, 30, 32].

The main outcomes of the serum vitamin D levels in the CSU patients compared to the controls are summarized at Table 2. Twelve studies showed statistically significantly lower levels of serum vitamin D in the CSU patients than the controls [14, 18, 20, 21, 23–25, 28, 29, 31, 33]. Wu et al. showed significantly higher levels of serum vitamin D in the CSU patients [30]. They compared the serum vitamin D levels of CSU patients in Southampton General Hospital to those of the general United Kingdom (UK) population (data from the National Diet and Nutrition Survey). The serum vitamin D levels of the 225 CSU patients were significantly higher than those of the 1321 UK population (control group). Lee et al. conducted a cross-sectional, population-based study of Korean children (aged 4–13 years; 3159 were controls; 624 had current urticaria, of which 57 were CSU and 567 acute urticaria). There was no statistically significant difference in the serum vitamin D levels of the CSU patients and the controls (p = 0.124) [22].

**Table 2:** Summary of parameters of vitamin D in CSU

Outcome measurement	Pro	Cons	Results	References
Lower serum vitamin D levels in CSU patients than healthy controls		✓	*One* study showed significantly higher levels of vitamin D in CSU patients than that of controls	Wu et al. [30]
	–	–	*One* study showed no significant difference in vitamin D levels between CSU patients and that of controls	Lee et al. [22]
	✓		*Twelve* studies showed significant lower levels of vitamin D in CSU patients than that of controls	Thorp et al. [28] Grzanka et al. [21] Chandrashekar et al. [20] Abdel-Rehim et al. [18] Movahedi et al. [23] Woo et al. [29] Rasool et al. [25] Boonpiyathad et al. [31] Oguz Topal et al. [24] Nasiri-Kalmarzi et al. [14] Dabas et al. [32] Rather et al. [33]
Vitamin D insufficiency in CSU patients more than in controls		✓	*One* study showed significantly higher prevalence of vitamin D insufficiency in controls than in CSU	Movahedi et al. [23]
		✓	*Two* studies showed no significant difference in the prevalence of vitamin D insufficiency between CSU patients and controls	Grzanka et al. [21] Boonpiyathad et al. [22] [31]
	✓		*One* study showed significant difference in the prevalence of vitamin D insufficiency between CSU patients and controls	Oguz Topal et al. [24]
Vitamin D deficiency in CSU patients more than in controls	–	–	*One* study showed no significant difference in the prevalence of vitamin D deficiency between CSU patients and controls	Thorp et al. [28]
	✓		*Three* studies showed significant difference in the prevalence of vitamin D deficiency between CSU patients and controls	Grzanka et al. [21] Boonpiyathad et al. [31] Oguz Topal et al. [24]
	✓		*One* study show significant difference in the proportion of critically low vitamin D levels in the CSU patients and in acute urticaria, atopic dermatitis, and healthy controls	Woo et al. [29]
Lower serum vitamin D levels between CSU and acute urticaria		✓	*One* study showed no significant difference levels of vitamin D between CSU and acute urticaria patients	Lee et al. [22]
	✓		*One* study showed significantly lower levels of vitamin D in CSU than acute urticaria patients	Woo et al. [29]
Lower serum vitamin D levels between CSU and atopic dermatitis	✓		*One* study showed significantly lower levels of vitamin D in CSU than atopic dermatitis	Woo et al. [29]
Lower serum vitamin D levels between CSU and allergic rhinitis	✓		*One* study showed significantly lower levels of vitamin D in CSU than allergic rhinitis	Thorp et al. [28]
Low serum vitamin D levels and higher disease activity		✓	*One* study reported a significant positive correlation between vitamin D levels and urticaria activity score	Movahedi et al. [23]
	–	–	*Six* studies reported no association	Thorp et al. [28] Abdel-Rehim et al. [18] Grzanka et al. [21] Rorie et al. [26] Boonpiyathad et al. [31] Oguz Topal et al. [24]
	✓		*Three* study reported significant negative association between vitamin D levels and urticaria activity score *One* study reported significant negative association between vitamin D levels and urticaria severity score	Woo et al. [29] Nasiri-Kalmarzi et al. [14] Rather et al. [33] Chandrashekar et al. [20]
Low serum vitamin D levels and longer disease duration	–	–	*Five* studies reported no association	Thorp et al. [28] Abdel-Rehim et al. [18] Grzanka et al. [21] Dabas et al. [32] Rather et al. [33]
	✓		*One* studies reported significant negative association	Woo et al. [29]
Low serum vitamin D levels and high ESR	✓		*Two* study reported significant correlation	Chandrashekar et al. [20] Boonpiyathad et al. [31]
Low serum vitamin D levels and high CRP levels	–	–	*One* study reported no association	Grzanka et al. [21]
Low serum vitamin D levels and high IgE levels	✓		*One* study reported negative association	Abdel-Rehim et al. [18]
	–	–	*Four* studies reported no association	Movahedi et al. [23] Oguz Topal et al. [24] Nasiri-Kalmarzi et al. [14] Dabas et al. [32]
Low serum vitamin D levels and high IL-17 levels	✓		*One* study reported negative association.	Chandrashekar et al. [20]
Low serum vitamin D levels and TGF-β1	✓		*One* study reported negative association	Chandrashekar et al. [20]
Low serum vitamin D levels and thyroid autoantibodies testing	–	–	*Two* studies reported no association	Thorp et al. [28] Oguz Topal et al. [24]
Low serum vitamin D levels and a positive ASST or APST	✓		*One* study reported significant lower levels of vitamin D in patients with a positive APST	Chandrashekar et al. [20]
	✓		*Three* study reported significant lower levels of vitamin D in patients with a positive ASST.	Woo et al. [29] Nasiri-Kalmarzi et al. [14] Rather et al. [33]
	–	–	*Three* studies reported no association between the ASST-positive and ASST-negative groups	Thorp et al. [28] Grzanka et al. [21] Dabas et al. [32]

APST, autologous plasma skin test; ASST, autologous serum skin test; CRP, C-reactive protein; CSU, chronic spontaneous urticaria; ESR, erythrocyte sedimentation rate; Ig, immunoglobulin; IL, interleukin; TGF-β1, transforming growth factor β1

### Degree of severity of serum vitamin D levels in CSU patients

The serum vitamin D levels were categorized into subgroups according to the vitamin D status. Serum 25(OH)D levels of > 30 ng/mL, 20–30 ng/mL, and < 20 ng/mL were defined as sufficiency, insufficiency, and deficiency, respectively; levels of < 10 ng/mL indicated a critically low or severe deficiency. The cut-point values to define vitamin D status in each study were very similar even though slightly different values were found in some studies (Table 3). The prevalence of vitamin D deficiency was reported more commonly in the CSU patients (34.3–89.7%) than in the controls (0.0–68.9%) in 8 studies [21, 23, 24, 28, 29, 31–33]. Four of those studies reported statistically significant differences [21, 24, 29, 31].

**Table 3:** Comparison of reported degree severity of serum vitamin D levels in CSU patients and controls

Studies	Thorp et al. [28]		Chandrashekar et al. [20]		Grzanka et al. [21]		Movahedi et al. [23]		Woo et al. [29]	
	Cases	Allergic rhinitis controls	Cases	Healthy controls	Cases	Healthy controls	Cases	Healthy controls	Cases	Healthy controls
N	25	25	45	45	35	33	114	187	72	72
Vitamin D levels	29.4 (mean)	39.6 (mean)	12.7 ± 2.7	24.3 ± 13.5	26.0 (median)	31.1 (median)	15.8	22.6	11.86 (mean)	20.77 (mean)
Sufficiency	ND	ND	ND	11/45 (24.44%)	ND	ND	10 (8.8%)	49 (26.2%)	2 (2%)	15 (20%)
Insufficiency	ND	ND	ND	18/45 (40%)	11 (31.4%)	13 (39.4%)	18* (15.8%)	31 (16.6%)	7 (10%)	20 (27%)
Deficiency	12 (48%)	7 (28%)	ND	16/45 (35.55%)	11* (31.4%)	2 (6%)	86 (75.4%)	107 (57.2%)	28 (39%)	32 (45%)
Severe deficiency	ND	ND	ND	ND	1 (2.9%)	0 (0%)	ND	ND	35* (49%)	5 (8%)
Definition										
Sufficiency	ND		> 30 ng/mL		≥ 30 ng/mL		ND		≥ 30 ng/mL	
Insufficiency	ND		Between 20 and 30 ng/mL		20–< 30 ng/mL		20–30 ng/mL		Between 20 and 29 ng/mL	
Deficiency	< 30 ng/mL		< 20 ng/mL		< 20 ng/mL		< 20 ng/mL		< 20 ng/mL	
Critically low/Severe deficiency	ND		ND		< 10 ng/mL		ND		< 10 ng/mL	

Studies	Rasool et al. [25]		Boonpiyathad et al. [31]		Oguz Topal et al. [24]		Nasiri-Kalmarzi et al. [14]		Rather et al. [33]	
	Cases	Healthy controls	Cases	Healthy controls	Cases	Healthy controls	Case	Healthy controls	Case	Controls
N	147	130	60	40	58	45	110	110	110	110
Vitamin D levels	17.87 (mean)	27.65 (mean)	15.0 (median)	30.0 (median)	8.45 (median)	15.3 (median)	19.26 ± 1.26 (mean)	31.72 ± 7.14 (mean)	19.6 ± 6.9 (mean)	38.5 ± 6.7 (mean)
Sufficiency	ND	ND	ND	ND	ND	ND	ND	ND	23 (20.91%)	71 (64.54%)
Insufficiency	91.3%	63.84%	28%	45%	57* (98.3%)	39 (86.7%)	58.02%	48.89%	17 (15.45%)	24 (21.82%)
Deficiency			55%*	0%	52* (89.7%)	31 (68.9%)			70 (63.64%)	15 (13.64%)
Severe deficiency	ND	ND	ND	ND	ND	ND	ND	ND	ND	ND
Definition										
Sufficiency	> 30 ng/mL		ND		> 30 µg/L		ND		> 30 ng/mL	
Insufficiency	20–30 ng/mL		> 20–< 30 ng/mL		< 30 µg/L		ND		20–30 ng/mL	
Deficiency	10–< 20 ng/mL		< 20 ng/mL		< 20 µg/L		ND		< 20 ng/mL	
Critically low/Severe deficiency	< 10 ng/mL		ND		ND		ND		ND	

CSU, chronic spontaneous urticaria; ND, not defined

* Significant difference compared to the control group

### Other effects of vitamin D on CSU

The effects of vitamin D on CSU are summarized at Table 2. The studies also compared the serum vitamin D levels of the CSU patients with those of patients with other diseases, such as acute urticaria [22, 29], atopic dermatitis [29], and allergic rhinitis [28]. Vitamin D level was significantly lower in CSU patients than in atopic dermatitis and allergic rhinitis [28, 29]. Four out of 11 studies reported significant association between low serum vitamin D levels and high disease activity whereas seven studies did not find this significant association. Most studies demonstrated that there was no association between low serum vitamin D levels and disease duration [18, 21, 28, 32, 33]. Others reported a relationship between the serum vitamin D levels and other investigations, including erythrocyte sedimentation rate [20, 31], C-reactive protein [21], serum IgE [14, 18, 23, 24, 32], IL-17 [20], transforming growth factor-β1 [20], thyroid autoantibodies [24, 28], autologous serum skin test [14, 21, 28, 29, 33], and autologous plasma skin test [20]. It was shown that low serum vitamin D level was significantly associated with high levels of ESR, IgE, IL-17, and transforming growth factor-β1 [18, 20, 31].

### Outcome of vitamin D supplementation on CSU patients

Seven studies (2 RCTs [26, 32], 3 case–control studies [24, 25, 31], 1 prospective study [19], and 1 case report [27]) were concerned with vitamin D supplementation in 587 CSU patients. The outcomes of the vitamin D supplementation were compared to baseline in 6 studies [19, 24–27, 32] and to controls in 1 study [31].

The regimens of vitamin D supplementation in each study were reviewed and are summarized at Table 4. Four studies used vitamin D_3_ at dosages ranging from 2800 to 75,000 IU/week [24–27], one study used vitamin D_2_ at a dosage of 140,000 IU/week [31], and another study did not define the form of vitamin D administered at a dosage of 50,000 IU/week [19]. Similarly, the form of vitamin D supplementation was also not defined in the RCT study but patients were categorized into three groups to receive low-dose (2000 IU/d), high-dose (60,000 IU/week), and without vitamin D supplementation, respectively [32]. The duration of the vitamin D supplementations ranged from 4 to 12 weeks. The serum vitamin D levels were evaluated in 4 studies and were reported as 25(OH)D [25–27, 31].

**Table 4:** Outcome of vitamin D supplement in CSU patients

Study, year	Study design	N	Enroll	Concomitant medications	Intervention (Dose, type, duration,source)	Duration	Main outcome measurement	Vitamin D status (ng/mL)		Outcome
								Before	End of treatment	
Sindher et al. [27]	Case report	1	Chronic urticaria	Calcium citrate 800 mg/day Fexofenadine Aluminium/magnesium antacid	Vitamin D3 (Cholecalciferol 400 IU/day	8 weeks	ND	4.7	ND	Continued to have intermittent urticaria
					Then increased to 2000 IU/day)	ND		ND	65	Complete resolution without antihistamine
Rorie et al. [26]	Prospective,double-blinded, randomized controlled trial (single-center clinical study)	42	CSU receiving high dose vitamin D3 (4000 IU/day) supplementation (n = 21)	Cetirizine Ranitidine Montelukast Use for intolerable or uncontrolled symptoms Prednisolone Hydroxychloroquine	Vitamin D3 4,000 IU/day	12 weeks	USS	*Vitamin D status* *(mean ± SE)*		*Decrease total USS scores* *(mean ± SE)*
								28.8 ± 2.2	56.0 ± 3.9	15.0 ± 2.9 (p = 0.02)
			CSU receiving low dose vitamin D3 (600 IU/day) supplementation (n = 21)		Vitamin D3 600 IU/day			37.1 ± 3.4	35.8 ± 2.3	24.1 ± 4.0
										Significant decrease in total USS score in the high, but not low, vitamin D3 treatment group by week 12 (p = 0.02) No correlation between 25(OH)D levels and USS score at baseline (r = 0.07, p = 0.65) or at week 12 (r = 0.13, p = 0.45) The high vitamin D3 treatment group showed a decreased total USS score compared with the low vitamin D3 treatment group, but this did not reach statistical significance (p = 0.052) Subjects in the high vitamin D3 treatment group reported decrease body distribution of hives on an average day (p = 0.033), decrease body distribution of hives on the worst day (p = 0.0085), and decrease number of days with hives (p = 0.03) compared with subjects in the low vitamin D3 treatment group.

Study, year	Study design	N	Enroll	Concomitant medications	Intervention (Dose, type, duration, source)	Duration	Main outcome measurement	Vitamin D status (ng/mL)		Outcome			
								Before	End of treatment				
Rasool et al. [25]	Randomized case–control study	147	CSU Any vitamin D levels (serum 25(OH)D) from *Group 1* Severe deficiency Vitamin D levels < 10 ng/mL *Group 2* Deficient levels Vitamin D levels 10–< 20 ng/mL *Group 3* Insufficient levels Vitamin D levels 20–30 ng/mL *Group 4* Sufficient levels Vitamin D levels > 30 ng/mL) Then randomized to Sub-group A (n = 48) sub-group B (n = 42) Sub-group C (n = 57)			6 weeks	VAS 5-D itch score	*Vitamin D status* *(mean ± SEM)*		*VAS score* *(mean ± SEM)*		*5-D itch score* *(mean)*	
								*Before*	*After*	*Before*	*After*	*Before*	*After*
				*Sub-group A*				*Sub-group A*		*Sub-group A*		*Sub-group A*	
				None	Vitamin D3 (cholecalciferol) 60,000 IU/week for 4 weeks			16.98 ± 1.43	56.74 ± 3.76 (p < 0 .0001)	6.7 ± 0.043	5.2 ± 0.70 (p = 0.0088)	14.5 ± 0.72	12.06 ± 1.10 (p = 0.0072)
				*Sub-group B*				*Sub-group B*		*Sub-group B*		*Sub-group B*	
				Hydroxyzine 25 mg/day for 6 weeks Corticosteroids (deflazacort) 6 mg/day for 6 weeks	None			17.04 ± 1.54	16.44 ± 1.50	6.6 ± 0.42	3.3 ± 0.50 (p < 0.0001)	13.9 ± 0.77	8.1 ± 1.13 (p < 0.001)
				*Sub-group C*				*Sub-group C*		*Sub-group C*		*Sub-group C*	
				Hydroxyzine 25 mg/day for 6 weeks Corticosteroids 6 mg/day for 6 weeks	Vitamin D3 60,000 IU/week for 4 weeks			18.95 ± 1.42	41.73 ± 2.85 (p < 0.0001)	6.68 ± 0.40	1.86 ± 0.39 (p < 0.0001)	13.9 ± 0.68	5.01 ± 0.94 (< 0.0001)
										Significantly decreased in VAS in every groups Significantly decreased in 5D itch score in every groups Improvement in the CSU symptoms in patients with vitamin D3 as monotherapy Better improvement of symptoms and quality of life in combinatorial therapy group than standard therapeutic regimen group Significant difference in VAS in subgroup A compared to subgroup B and C (p = 0.016 and p < 0.0001, respectively) Significant difference in VAS in subgroup C compare to subgroup B (p = 0.0203) Significant difference in 5-D score in subgroup A compared to subgroup B and C (p = 0.0116 and p < 0.0001, respectively) Significant difference in 5-D score in subgroup C compared to subgroup B (p = 0.0382)			
		130	Healthy control	None	None	6 weeks	Vitamin D levels	*Group 1*		No change in serum 25(OH)D levels			
								7.310 ± 0.52	5.899 ± 0.28				
								*Group 2*					
								15.26 ± 0.47	16.96 ± 1.26				
								*Group 3*					
								23.98 ± 0.46	23.15 ± 0.95				
								*Group 4*					
								47.78 ± 2.23	49.18 ± 2.97				
Oguz Topal et al. [24]	Prospective case–control study	57 cases	CSU Serum 25(OH)D < 30ug/L	None	Vitamin D3 300,000 IU/month	12 weeks	UAS4^‡‡^ CU-Q2oL	ND	ND	*UAS4* *(median(min–max))*		*CU-Q2oL* *(median(min–max))*	
										*Before*	*After*	*Before*	*After*
										21 (0–42.0)	6 (0–21.0) (p < 0.001)	38 (6.5–115.2)	10.8 (0–43.4) (p < 0.001)
										Significant improvements in UAS4 and CU-Q2oL			
Boonpiyathad et al. [31]	Prospective case–control study	50 cases	CSU Serum 25(OH)D < 30 ng/mL (vitamin D supplement group)	Non-sedative antihistamine	Ergocalciferol (vitamin D2) 20,000 IU/day	6 weeks	UAS7 DLQI	13 (8–29) median (min–max)	40 (28–62) median (min–max)	*UAS7*		*DLQI scores*	
										*Before*	*After*	*Before*	*After*
										27 (6–38)	15 (2–33)	13 (4–31)	6 (1–20)
		10 controls	CSU Serum 25(OH)D ≥ 30 ng/ml (non-vitamin D supplement group)	ND	None	6 weeks	UAS7 DLQI	37 (33–52) median (min–max)	38 (33–52) median (min–max)	26 (18–42)	26 (16–44)	12 (5–28)	14 (3–27)
										Significant improvements in UAS7 and DLQI scores in the vitamin D supplement group compared with the non-vitamin D supplement group Significant improvement of the median UAS7 score in the vitamin D supplement group than in the non-vitamin D supplement group Significantly improvement of the median DLQI score in the vitamin D supplement compared with the non-vitamin D supplement group None of the patients in the vitamin D supplement group were symptom-free at the optimal vitamin D levels.			
Ariaee et al. [19]	Prospective study	20	CSU Serum vitamin D concentration < 10 ng/mL	ND	Vitamin D 50,000 unit/week	8 weeks	USS DLQI	ND	ND	*USS (mean ± SD)*		*DLQI scores (mean ± SD)*	
										*Before*	*After*	*Before*	*After*
										235 ± 13.9	11.2 ± 9.6	10.8 ± 1.6	0.9 ± 4.8
										Significant reduction in USS after vitamin D supplement Improvement of DLQI (55%) after vitamin D supplement Increase FOXP3 gene expression and downregulation of IL-10, TGF-beta and FOXP3, IL-17 after vitamin D supplement			
Dabas et al. [32]	Randomized controlled trial	200	CSU Serum 25(OH)D < 30 nmol/L	Levocetirizine 10 mg/day	*Group A* Vitamin D 2000 IU/day *Group B* Vitamin D 60,000 IU/week *Group C* None	12 weeks	UAS4	ND	ND	*UAS4 (mean)*			
										*Before*	*After 6* *weeks*	*After 12* *weeks*	
										*Group A*			
										11.8 ± 7.6	6.6 ± 6.0	5.3 ± 5.2	
										*Group B*			
										13.0 ± 8.0	6.4 ± 5.0	4.2 ± 3.5	
										*Group C*			
										12.9 ± 7.03	8.0 ± 5.7	6.1 ± 4.8	
										No significant difference in mean UAS4 in the 3 groups after 12 weeks of vitamin D replacement Vitamin D replacement decreased the severity in most patients.			

25(OH)D, 25-hydoxyvitamin D; 5-D itch score, 5-dimension itch score; CSU, chronic spontaneous urticaria; CU-Q2oL, Chronic Urticaria Quality of Life Questionnaire; DLQI, Dermatology Life Quality Index; IL, interleukin; TGF, transforming growth factor; ND, not defined; UAS, urticaria activity score; USS score, the Urticaria Symptom Severity Score; VAS, visual analogue scale

^‡‡^UAS4 (the Urticaria Activity Score over 4 days; (scale 0–6) calculated as the sum of daily average morning and evening scores for itch severity (0, none; 1, mild; 2, moderate; 3, severe) and number of hives (0, none; 1, < 20 hives; 2, 20–50 hives; and 3, > 50 hives)

The parameters of treatment outcomes varied among the studies; they comprised the urticaria activity score over 4 days (UAS4) [24, 32], urticaria activity score over 7 days (UAS7) [31], dermatology life quality index [19, 31], chronic urticaria quality of life questionnaire [24], visual analogue scale [25], 5-dimension itch score [25], and urticaria symptom severity score [19, 26] (Table 5). Four studies reported a significant reduction in disease activity after high dose vitamin D supplementation (vitamin D_2_, 140,000 IU/week; vitamin D_3_, 60,000–75,000 IU/week; and unknown form of vitamin D, 50,000 unit/week) [19, 24, 25, 31]. One case report showed that treatment with a low vitamin D dosage (400 IU/d) for 2 months did not reduce urticaria activity. However, complete resolution without antihistamine was demonstrated at a higher dosage (2000 IU/d) [27]. Another study reported a significant reduction in disease activity after high-dose vitamin D supplementation (4000 IU/d) compared to low-dose vitamin D supplementation (600 IU/d) [26]. Ariaee et al. reported that the transforming growth factor-β, IL-10 and IL-17 expressions were decreased after 8 weeks of vitamin D supplementation [19]. In addition, forkhead box P3 (FOXP3) expression, a clinical determinant of Treg, increased after treatment [19]. In the RCT study, either low-dose or high-dose of vitamin D supplementation could reduce disease severity but there was no significant difference in the mean UAS4 among the three groups after 12 weeks of supplementation [32].

**Table 5:** Summarized of treatment regimens and outcome of vitamin D supplementation

N	Sindher et al. [27]**		Rorie et al. [26]^††^		Rasool et al. [25]^§§^				Oguz Topal et al. [24]		Boonpiyathad et al. [31]		Ariaee et al. [19]		Dabas et al. [32]		
	1		21	21	48		57		57		50		20		200		
Intervention	Vitamin D3 400 IU/day	Vitamin D3 2000 IU/day	Vitamin D3 4000 IU/day	Vitamin D3 600 IU/day	Vitamin D3 60,000 IU/week		Vitamin D3 60,000 IU/week, 4 weeks Hydroxyzine 25 mg/day, 6 weeks Corticosteroid 6 mg/day, 6 weeks		Vitamin D3 300,000 IU/month		Vitamin D2 20,000 IU/day		Vitamin D (unknown form) 50,000 unit/week		Vitamin D (unknown form) *Group A* Vitamin D 2000 IU/day *Group B* Vitamin D 60,000 IU/week *Group C* None		
Duration	8 weeks	ND	12 weeks	12 weeks	4 weeks		4 weeks		12 weeks		6 weeks		8 weeks		12 weeks		
*Vitamin D status (ng/mL)*																	
Before treatment	4.7	ND	28.8 ± 2.2	37.1 ± 3.4	16.98 ± 1.43		18.95 ± 1.42		ND		13 (8–29) median (min–max)		ND		ND		
End of treatment	ND	65	56.0 ± 3.9	35.8 ± 2.3	56.74 ± 3.76 (p < 0 .0001)		41.73 ± 2.85 (p < 0.0001)		ND		40 (28–62) median (min–max)		ND		ND		
Outcome	Continued to have intermittent urticaria	Complete resolution without antihistamine	*Decrease total USS scores* *(mean ± SE*)		*VAS score* *(mean ± SEM)*				*UAS4* *(median(min–max))*		*UAS7*		*USS* *(mean ± SD)*		*UAS4 (mean)*		
			15.0 ± 2.9 (p = 0.02)	24.1 ± 4.0	*Before*	*After*	*Before*	*After*	*Before*	*After*	*Before*	*After*	*Before*	*After*	*Before*	*After 6* *weeks*	*After 12* *weeks*
					6.7 ± 0.04	5.2 ± 0.70 p = 0.009	6.68 ± 0.40	1.86 ± 0.39 p < 0.0001	21 (0–42.0)	6 (0–21.0) p < 0.001	27 (6–38)	15 (2–33)	235 ± 13.9	11.2 ± 9.6	*GroupA*		
															11.8 ± 7.6	6.6 ± 6.0	5.3 ± 5.2
					*5-D itch score* *(mean)*				*CU-Q2oL* *(median(min–max))*		*DLQI scores*		*DLQI scores* *(mean ± SD)*		*Group B*		
					*Before*	*After*	*Before*	*After*	*Before*	*After*	*Before*	*After*	*Before*	*After*	13.0 ± 8.0	6.4 ± 5.0	4.2 ± 3.5
					14.5 ± 0.72	12.06 ± 1.10 p = 0.007	13.9 ± 0.68	5.01 ± 0.94 p < 0.0001	38 (6.5–115.2)	10.8 (0–43.4) p < 0.001	13 (4–31)	6 (1–20)	10.8 ± 1.6	0.9 ± 4.8	*Group C*		
															12.9 ± 7.03	8.0 ± 5.7	6.1 ± 4.8

5-D itch score, 5-dimension itch score; CU-Q2oL, Chronic Urticaria Quality of Life Questionnaire; DLQI, Dermatology Life Quality Index; ND, not defined; UAS, urticaria activity score; USS score, the Urticaria Symptom Severity Score; VAS, visual analogue scale

**CSU patients in Sindher et al. was treated with low dose vitamin D3 without response, then the patient was treated with higher dosage of vitamin D3 [26]

^††^CSU patients in Rorie et al. were randomized to vitamin D3 4000 IU/day or 600 IU/day [25]

^§§^CSU patients in Rasool et al. were randomized to vitamin D3 60,0000 IU/week alone or vitamin D3 60,000 IU/week and hydroxyzine 25 mg/day and corticosteroid 6 mg/day [24]

## Discussion

Two recent meta-analysis regarding the association between vitamin D and urticaria have been published in 2018. Tsai et al. and Wang et al. showed that the prevalence of vitamin D was significantly higher in CU patients than that of controls. [34, 35] Similar to those two meta-analysis, 12 out of 14 studies in our study showed significantly lower levels of serum vitamin D in CSU patients than in the controls [14, 18, 20, 21, 23–25, 28, 29, 31, 33]. Only Wu et al. found significantly higher levels of vitamin D in the CSU patients than in the UK general population as a control group [30]. However, that study compared CSU patients in Southampton General Hospital to the UK general population rather than healthy controls in Southampton; a variation of serum vitamin D levels in different regions of UK was reported [36]. Lee et al. [22] reported no statistical significance between the vitamin D levels in pediatric CSU patients and the controls, which was similar to a study by Tsai et al. [34]. Nevertheless, it should be noted that our study provides additional information regarding associations between vitamin D and urticaria than those of the two studies. Data regarding (1) types of serum vitamin D (2) outcome of vitamin D supplementation after treating with different dosages, types and duration of vitamin D are also added in this study.

Potential factors determining vitamin D status include oral vitamin D intake, sun exposure, latitude, season, Fitzpatrick skin type, time spent outdoors, sun exposure practices, body mass index (BMI), physical activity, alcohol intake, and genetic polymorphism [37]. Higher serum vitamin D levels can be observed with prolonged sun exposure, increased time spent outdoors, the summer season, living in lower latitudes, increased physical activity, moderate alcohol intake, and rs7041 gene polymorphism [37]. In contrast, lower serum vitamin D levels can be observed with darker skin, female gender, higher BMI, excessive alcohol intake, and rs4588 gene polymorphism [37]. It has been reported that vitamin D deficiency and insufficiency is a pandemic problem. The prevalence of vitamin D deficiency and insufficiency has been estimated to be 30%-60% of children and adults worldwide. Areas that had high prevalence of vitamin D deficiency and insufficiency in the general population were Europe (92%), Middle East (90%), Asia (45–98%), and Canada (61%). The most common cause of vitamin D deficiency and insufficiency is an insufficient exposure to sun-light as diet with fortified vitamin D are few. For example, in Middle East, vitamin D deficiency is found to strongly correlate with well-covering clothes [38, 39].

Vitamin D has been shown to be linked to other skin diseases. Low serum 25(OH)D levels have been reported in severe atopic dermatitis [40], psoriasis [41], vitiligo [42], systemic sclerosis [43], severe alopecia areata [44], severe systemic lupus erythematosus (SLE) [45], and acne [46] and also associated with an increased risk of cutaneous bacterial infections in vitro [47]. However, no studies in our review reported the cut-off serum vitamin D levels that might be associated with the development of CSU.

As to vitamin D supplementation, both vitamins D_2_ and D_3_ are commonly. Current dietary reference intakes for vitamin D are 400 IU per day in infancy, 600 IU per day in the 1–70 year age group, and 800 IU per day for individuals aged over 70 [48]. Vitamin D_2_ is reported to be less effective than vitamin D_3_ in raising total serum vitamin D levels, but less toxic than vitamin D_3_ when given in large amounts [2]. The variations in the vitamin D supplementation regimens in the studies might have led to different outcomes.

Six studies showed that a high dosage of vitamin D treatment resulted in a significant reduction in CSU activity. [19, 24–27, 31] The other study reported that vitamin D supplement 2000 IU/day and 60,000 IU/week decreased disease activity in most CSU patients [32].

Among the various regimens, higher dosages of vitamin D (vitamin D_3_ of at least 28,000 IU/week for 4–12 weeks, or vitamin D_2_ of 140,000 IU/week for 6 weeks) were reported to be effective. Although the available studies were relatively scarce, CSU patients with low serum vitamin D levels at baseline tended to show an improvement after receiving high dose vitamin D supplementation. Vitamin D has high safety margin. The tolerable upper intake levels are now 4000–10,000 IU/d for adults and the elderly, and lower for infants and young children [48, 49]. According to our systematic review, even though there were not reported any adverse effect during vitamin D therapy, high dosage of vitamin D use should be concerned about safety. Measurement of serum vitamin D levels may be useful for safety monitoring and determining relationship to the treatment outcome, and it should be concerned about potential adverse effect at serum 25(OH)D levels greater than 50 ng/ml (125 nmol/liter) [48].

Vitamin D supplementation was reported for other skin diseases. A meta-analysis by Kim et al. of 4 randomized, double-blind, placebo-controlled trials showed that the SCORAD index and EASI score of atopic dermatitis patients decreased significantly after vitamin D supplementation [50]. Lim et al. compared the vitamin D levels of patients with and without acne in a case–control study combined with a randomized controlled trial [46]. Improvements in inflammatory lesions were noted after vitamin D supplementation in 39 acne patients with 25(OH)D deficiency. Abou–Raya et al. randomized 267 patients with SLE to receive either vitamin D_3_ (2000 IU daily) or a placebo. At 12 months of treatment, there was a significant decrease in the pro-inflammatory cytokines levels (i.e., IL-1, IL-6, IL-18 and TNF-α), anti-dsDNA, C4, fibrinogen, von Willebrand factor, and disease activity scores of the treatment group compared to the placebo group [51].

This systematic review has some limitations. First, there are small numbers of relevant studies. Second, few studies are RCTs; and variety in the individualized vitamin D supplementation regimens contribute to unsettle treatment results.

## Conclusions

Most studies showed that CSU patients had significantly lower serum vitamin D levels than the controls [14, 18, 20, 21, 23–25, 28, 29, 31–33]. However, this relationship does not prove causation. Data from a limited number of studies showed that the responders tended to be CSU patients with low serum vitamin D at baseline who received high-dose vitamin D supplementation regimens. For recalcitrant CSU patients with low serum vitamin D levels, a high dose of vitamin D supplements for 4–12 weeks may be used as an adjunctive treatment. Well-designed randomized placebo-controlled studies should be performed to determine the cut-off levels for vitamin D supplementation and treatment outcomes.

## Abbreviations

1,25(OH)_2_D: 1,25-dihydroxyvitamin D
25(OH)D: 25-hydroxyvitamin D
BMI: body mass index
CIU: chronic idiopathic urticaria
CSU: chronic spontaneous urticaria
CU: chronic urticaria
GC: group-specific component
IL: interleukin
RCT: randomized controlled trial
SLE: systemic lupus erythematosus
SNPs: single nucleotide polymorphisms
Treg cells: regulatory T cells
UAS: urticaria activity score
UK: United Kingdom
VDBP: vitamin D binding protein
VDR: vitamin D receptor
